# Terminal differentiation of human granulosa cells as luteinization is reversed by activin-A through silencing of Jnk pathway

**DOI:** 10.1038/s41420-020-00324-9

**Published:** 2020-09-23

**Authors:** Gamze Bildik, Nazli Akin, Yashar Esmaeilian, Francesko Hela, Ceren Sultan Yildiz, Ece Iltumur, Said İncir, Sercin Karahuseyinoglu, Kayhan Yakin, Ozgur Oktem

**Affiliations:** 1 The Graduate School of Health Sciences, Koc University, 34450 Istanbul, Turkey; 2 Department of Experimental Therapeutics, The University of Texas MD Anderson Cancer Center, Houston, TX 77030 USA; 3 Follicle Biology Laboratory (FOBL), Department of Pathology/Molecular and Cellular Medicine, Vrije Universiteit, 1050 Brussel, Belgium; 4 Research Center for Translational Medicine, Koc University, 34450 Istanbul, Turkey; 5 Department of Clinical Biochemistry and Laboratory, Koc University Hospital, 34450 Istanbul, Turkey; 6 Department of Histology and Embryology, Koc University School of Medicine, 34010 Istanbul, Turkey; 7 Department of Obstetrics and Gynecology, Koc University Hospital and School of Medicine, 34010 Istanbul, Turkey

**Keywords:** Hormone receptors, Translational research

## Abstract

Molecular mechanisms underlying luteinization (terminal differentiation of granulosa and theca cells after ovulation) and luteolysis (demise of corpus luteum) are poorly understood in human ovary. Here we report that activin-A, after binding to its cognate receptors induces a functional luteolytic state and reverses luteinization phenotype by downregulating the expression of the steroidogenic enzymes, LH receptor and VEGF and reducing estradiol (E_2_) progesterone (P_4_) production and upregulating FSH receptor and cyclin D1 expression in human primary luteinized granulosa cells. Further, this action of activin-A involves downregulation of JNK signaling pathway and is opposite to that of human chorionic gonadotropin (hCG), which acts as a luteotropic hormone and improves luteal function through the activation of JNK pathway in the same cell type. Reversal of luteinization phenotype in luteal granulosa cells by activin-A potentially makes this hormone an attractive candidate for use under certain clinical situations, where induction of luteolysis and rapid reduction of endogenous sex steroid levels are beneficial such as ovarian hyperstimulation syndrome (OHSS), in which the ovaries hyper-respond to gonadotropin stimulation by producing too many growing follicles along with development of ascites, pleural effusion, and hemo-concentrations as a result of increased vascular permeability and leakage of intravascular volume into third spaces. Our work unveils a previously undefined role for activin-A and JNK signaling pathway in human corpus luteum biology, that might have a direct clinical impact in assisted reproductive technologies.

## Introduction

Corpus luteum is a transient endocrine organ that is composed of different compartments and cell types^1^. It is the main source of progesterone production during luteal phase of the menstrual cycle that is required for the preparation of endometrium for implantation. If pregnancy occurs CL is rescued from undergoing demise (luteolysis) and continues to produce progesterone (P_4_) hormone by the stimulatory action of human chorionic gonadotropin (hCG) produced by the trophoblasts of the embryo until luteo-placental shift occurs and placenta becomes the major source of P_4_ production for the remainder of pregnancy^2^. Luteinization process and luteolysis are complex event that are poorly understood in human. While the former is characterized by a marked tissue remodeling, marked changes in intracellular signaling, gene regulations, extracellular matrix adhesion proteins, angiogenesis, cell cycle regulators^3–5^ the latter can be defined as loss of function, decrease in P synthesis and output, tissue break-down, degradation of extracellular matrix proteins, infiltration of macrophages, and subsequent involution of the luteal structures and vessels and increased cell death^1,6^.

There are three important issues regarding the luteal function in human assisted reproduction as follows: First, it is well documented that the functions of corpus luteum are defective in patients who undergo ovarian stimulation with gonadotropin hormones during assisted reproduction^7^. This necessitates progesterone and/or hCG administration known as luteal phase support in order to improve clinical pregnancy rates and prevent miscarriage^8,9^. Second important issue is the risk of ovarian hyperstimulation syndrome (OHSS) during assisted reproduction in which the ovaries hyper-respond to gonadotropin stimulation by producing too many growing follicles along with development of ascites, pleural effusion, and hemoconcentrations as a result of increased vascular permeability and leakage of intravascular volume into third spaces^10–12^. As a third issue there are some clinical circumstances in which early luteolysis and rapid reduction in serum estrogen level are indicated such as IVF patients with estrogen sensitive tumors such as breast cancer^13^. Therefore, identification of hormones signaling pathways involved in the luteinization and luteolysis are of paramount importance from the perspective of clinical practice in IVF.

Activin-A is a member of TGF-β superfamily and has a number of actions defined in human reproductive functioning such as stimulation of the production of FSHβ subunit from the gonadotropes in the anterior pituitary, mitogenic effect on granulosa cells (GCs) of the growing follicles, upregulation of their FSH receptor expression and oocyte maturation^14,15^. It was also documented that GCs continue to express activin-A and its cognate receptors after luteinization process^15^. However, available data is limited and somewhat controversial regarding the biological role of activin-A in these cells. This works unveils a novel mechanism that link activin-A and JNK signaling pathway in corpus luteum biology using human luteal granulosa cells obtained from IVF patients undergoing assisted reproduction.

## Results

### Validation experiments

It was previously documented that human luteal granulosa cells (GCs) continue to have steroidogenic activity in vitro^16–22^. In agreement, we first confirm that these cells have a certain basal steroidogenic activity that produce detectable amounts of E_2_ and P_4_ even under serum-free culture condition. When fetal bovine serum (FBS) was added to the culture medium, E_2_ and P_4_ output of the cells were further enhanced in proportion with increasing concentration of FBS (Supplementary Figs. 1A and 1B). Next, we analyzed and validated the expression of activin receptors type 1 and type 2 (ACVR1, ACR2) in these cells with qRT-PCR method (Supplementary Fig. 1C). Treatment of the cells with activin-A at 5, 25, and 100 ng/mL concentrations for 30 min induced phosphorylation of Smad2 as shown by a dose-dependent increase in the expression of phospho-Smad2^Ser465/Ser467^ in western blotting. This effect was abolished when the cells were treated with activin-A and a receptor blocker (Supplementary Fig. 1D). Taken together, these validation experiments confirm as a proof of concept the presence of the steroidogenic function, the expression of activin receptors and their responsiveness to exogenously administered activin-A in human luteal GCs in vitro.

### Activin-A downregulates the expression of steroidogenic enzymes and reduces E_2_ and P_4_ production in human luteal GCs

**Fig. 1:**
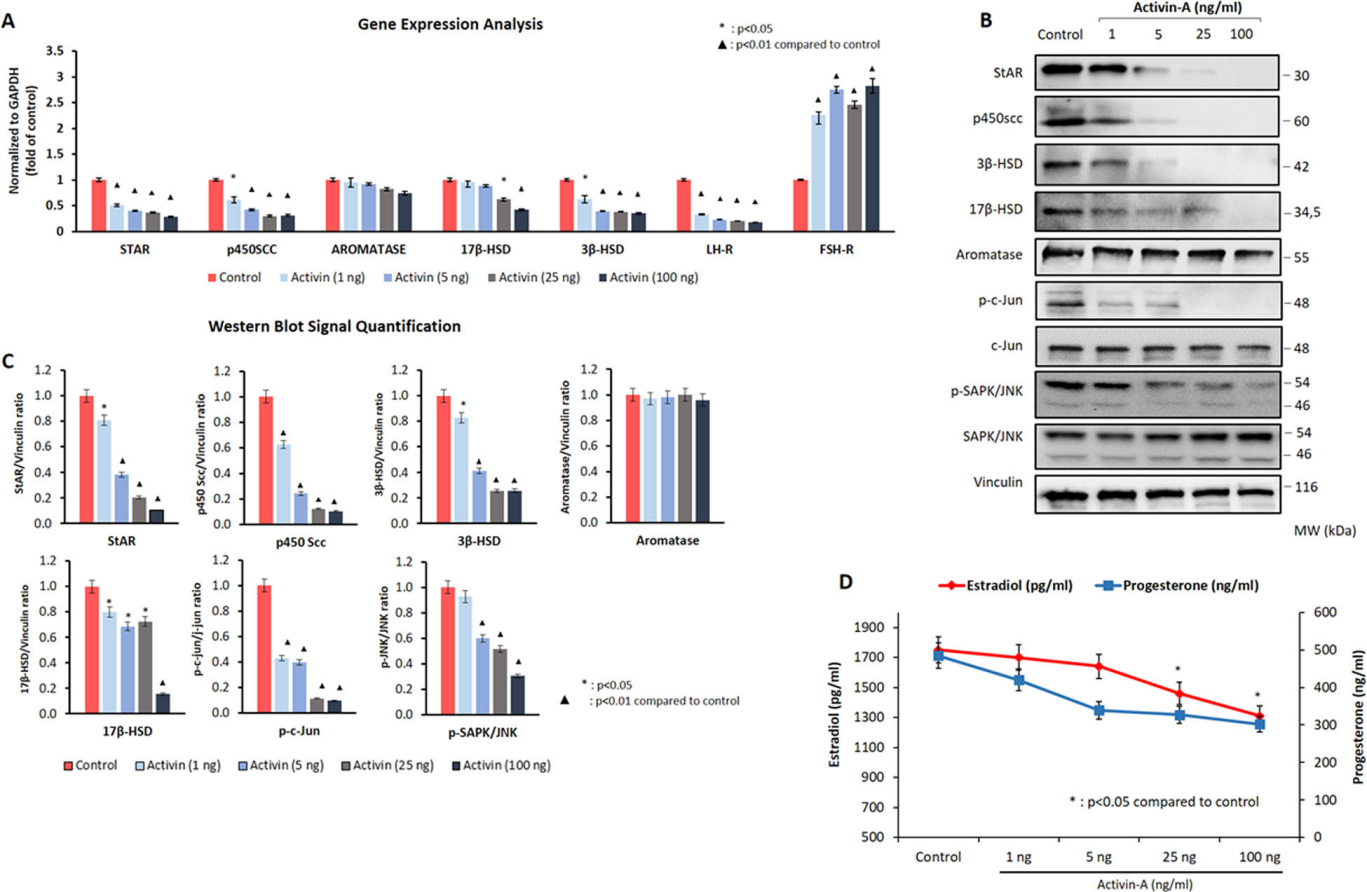
The effect of activin-A on the luteal steroidogenic function and JNK expression in human luteal granulosa cells. Activin-A treatment is associated with significant decrease in the expression of steroidogenic enzymes StAR, P450 SCC, 3β-HSD, and 17β-HSD on qRT-PCR (**a**) and western blotting (**b**, **c**) along with decreased production of E_2_ and P_4_ (**d**). Note that aromatase expression did not change meaningfully after activin-A treatment either at transcriptional or translational level. The expression of phospho-c-Jun^Ser73^ and phospho-JNK^SerThr183/Tyr185^ expression were gradually diminished along with the steroidogenic enzymes in western blotting (**b**, **c**). FSH receptor expression was significantly upregulated and LH receptor was down-regulated in the cells in a dose-dependent fashion after activin-A treatment on qRT-PCR (**a**).

Having confirmed the expression and biological activity of activin receptors in human luteal GCs, we next analyzed the effect of activin-A on the expression of the steroidogenic enzymes, gonadotropin receptors, and hormone production in these cells. Incubation of the cells with activin-A at four different concentrations (1, 5, 25, and 100 ng/ml) for 24 h resulted in a significant downregulation in the expression of the steroidogenic enzymes StAR, P450 SCC, 3β-HSD, and 17β-HSD in a dose-dependent fashion as shown both on qRT-PCR (Fig. 1a) and western blotting (Fig. 1b, c). However, such a notable change was not observed in the aromatase expression after activin-A treatment either at transcriptional or translational level (Fig. 1a, b). In line with downregulated expression of the steroidogenic enzymes, in vitro E_2_ and P_4_ production of the cells gradually decreased following treatment with activin-A at incremental concentration (Fig. 1d). We also observed that the suppressive effect of activin-A on the steroidogenic function of the cells persisted and their E_2_ and P_4_ production continued to decrease progressively when culture period was extended up to 96 h (Supplementary Fig. 2).

In order to rule out the possibility that declining E_2_ and P_4_ production of the cells after activin-A treatment is due to reduced viability rather than suppression of steroidogenic activity, we compared the viability of the cells using intravital carbocyanine uptake assay, and could not find any difference in the vitality of the cells before and after activin-A treatment (Supplementary Fig. 3).

Since these results were obtained in the luteal GCs retrieved from the patients stimulated with GnRH antagonist protocol, we repeated the experiments in the GCs of natural cycle and GnRH agonist stimulation protocol and obtained similar findings (Supplementary Fig. 4).

We also observed that FSH receptor expression was significantly upregulated and LH receptor was down-regulated in the cells in a dose-dependent fashion after activin-A treatment on qRT-PCR (Fig. 1a). Increased vascularization and decreased expression of cyclin D1 are other hallmarks of luteinization in the granulosa cells^5,6,23^. We therefore compared the expression of these markers in another set of experiments and found that activin-A significantly downregulated the expression of LH receptor, VEGF and upregulated FSH receptor and cyclin D1, while hCG as a luteotropic hormone exerted the opposite effects (Supplementary Fig. 5).

### Activin-A downregulates the steroidogenic enzymes along with JNK pathway in human luteal granulosa cells

**Fig. 2:**
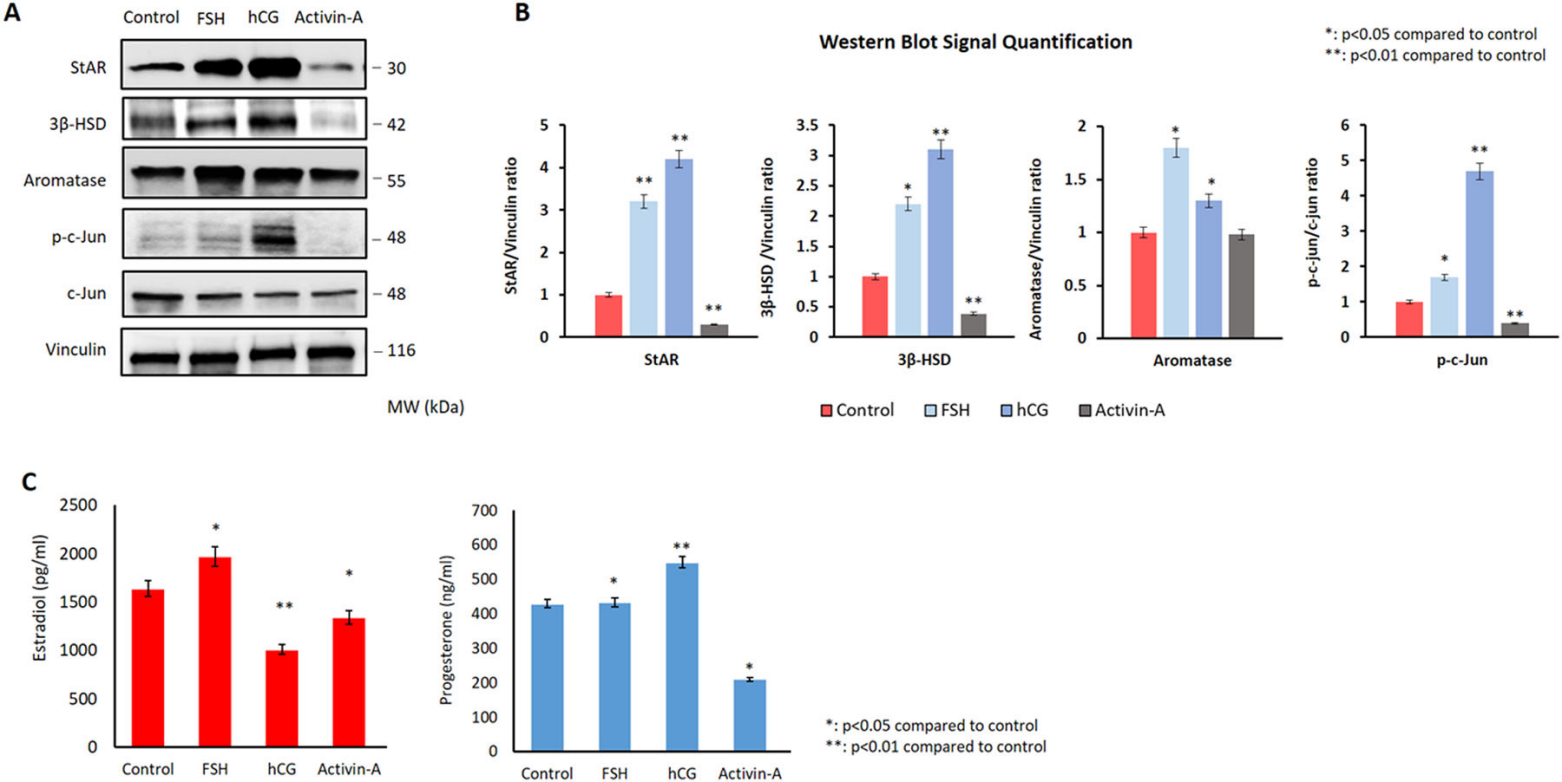
hCG and Activin-A have opposing effects on the luteinization phenotype in human luteal granulosa cells. hCG treatment (10IU/ml) significantly upregulated StAR, 3β-HSD expression along with phospho-c-Jun (**a**, **b**) in western blotting and increased P_4_ output in the cells (**c**). By contrast, activin-A (25 ng/ml) exerted opposite effects and reduced P_4_ production. FSH (25 mIU/ml) increased the expression of aromatase (**a**, **b**) and increased E_2_ production (**c**) but did not change the levels of total or phosphorylated c-Jun.

**Fig. 3:**
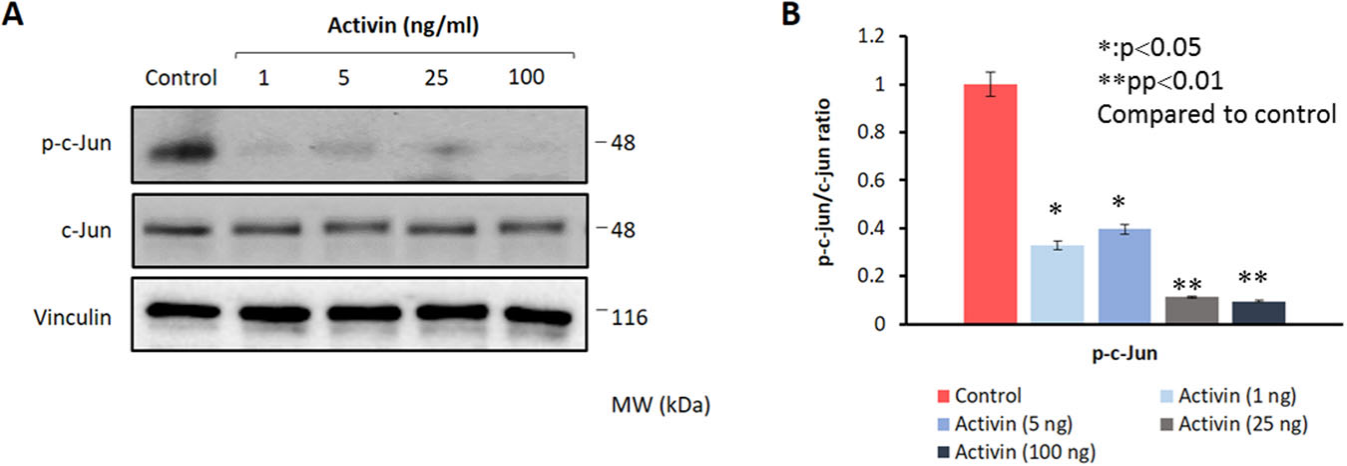
Activin-A reduces endogenous kinase activity of JNK in human luteal granulosa cells. Activin-A treatment caused a dose-dependent decrease in the kinase activity of JNK as evidenced by decreased phosphorylation of c-Jun^Ser63^. Total c-Jun expression remained stable. Vinculin is used as a loading control.

Our findings so far provided supporting evidence that activin-A treatment reverses luteinization phenotype in the luteal GCs, as evidenced by decreased expression of steroidogenic enzymes particularly StAR and 3β-HSD, LH receptor, VEGF-A, cyclin D1, and reduced E_2_/P_4_ production. We have recently shown in another study that hCG improves luteal function by upregulating the expression of steroidogenic enzymes and enhancing P_4_ output through activation of JNK pathway in human luteal GCs^24^. Furthermore, we also observed in that study that hCG stimulation of the cells after silencing of JNK via siRNA failed to improve the luteal defect and increase P_4_ output, suggestive of a pivotal role for JNK in the regulation of steroidogenesis in these cells. Given that hCG and activin-A have opposed effects on the luteinization characteristics of the GCs, we therefore, hypothesized if hCG induced the improvement of luteal function involves upregulation of JNK pathway in luteal GCs, activin-A induced reversal of luteinization might involve downregulation of JNK pathway in these cells. To test our hypothesis, we conducted a series of experiment. First, we simultaneously analyzed the effect of hCG and activin-A on the steroidogenic function and JNK pathway in these cells. As shown in the Fig. 2, hCG (10 IU/ml) significantly upregulated the expression of StAR, 3β-HSD, and phospho-c-Jun along with increased P_4_ production of the cells. By contrast, activin-A (25 ng/ml) produced opposite effects on these cells by downregulating the expression of these enzymes, phospho-c-Jun and reducing their P_4_ production. When the cells were treated with activin-A at different concentrations (1, 5, 25, and 100 ng/ml) rather than a single dose we obtained the same findings. The expression of phospho-c-Jun^Ser73^ and phospho-JNK^SerThr183/Tyr185^ were diminished gradually. However total JNK and c-Jun expression did not change, suggestive of a change in the JNK activity after activin-A (Fig. 1b, c). To confirm this observation, we next carried out a kinase assay and found that endogenous kinase activity of JNK was diminished in the cells treated with activin-A as evidenced by reduced phosphorylation of recombinant c-Jun at Ser73 residue (Fig. 3a, b).

**Fig. 4:**
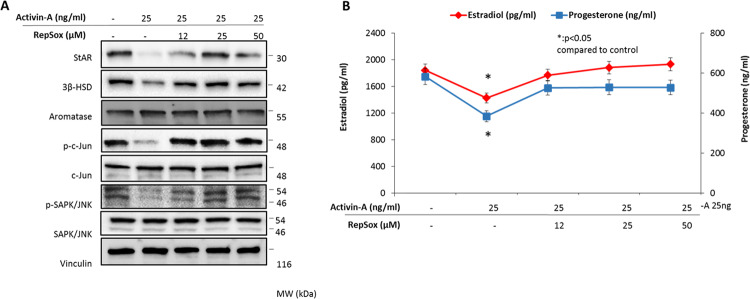
Antagonizing the actions of activin-A with a receptor blocker relieves its suppressive effects on steroidogenesis and JNK pathway in human luteal granulosa cells. When the biological actions of activin-A was inhibited with a selective receptor blocker the suppressive effects of activin-A on the steroidogenic enzymes and JNK pathway were reversed and the expression of StAR, 3β-HSD, phospho-c-Jun^Ser73^, and phospho-JNK^SerThr183/Tyr185^ were increased along with E_2_ and P production of the cells (**a**, **b**). Aromatase expression did not change meaningfully after activin-A treatment with and without the receptor blocker (**a**).

In another set of experiments, the cells were treated with activin-A with and without a receptor blocker to analyze the impact of blocking activin-A signaling on the steroidogenic function and JNK expression. We observed that the suppressive effects of activin-A on the steroidogenic enzymes and JNK pathway were relieved and the expression of StAR, 3β-HSD, phospho-c-Jun^Ser73^, and phospho-JNK^SerThr183/Tyr185^ increased together with increased E_2_ and P_4_ production of the cells when the biological actions of activin-A was antagonized with a selective receptor blocker (Fig. 4a, b).

**Fig. 5:**
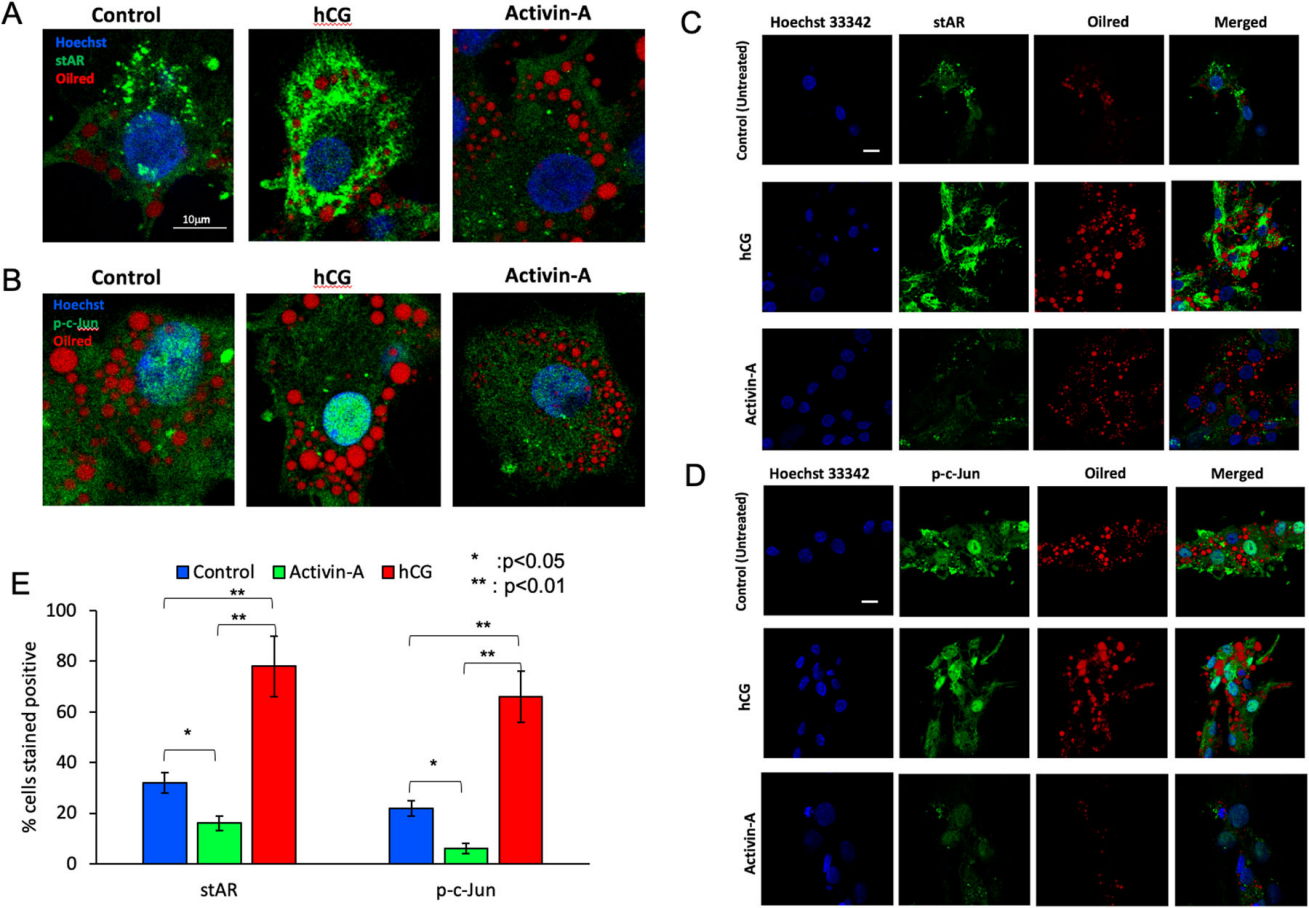
Confocal images of the luteal granulosa cells. The intensity of staining for StAR (green signal, **a** high magnification, **c** low magnification) and phospho-c-Jun (green signal, **b** high magnification, **d** low magnification) are substantially increased and decreased after treatment with hCG and activin-A respectively. In accordance with this the percentage of the cells stained positive for these markers were significantly decreased in the activin-treated cells in comparison to control and hCG-treated ones (**e**). Nuclei were highlighted in blue with DAPI staining. Oil Red O staining shows neutral lipid droplets in the cells as red dots. Scale bar is 10 μm.

Consistent with western blot results, the confocal images showed that the percentages of the cells immuno-stained positively for StAR and phospho-c-Jun^Ser73^ were significantly decreased after treatment with activin-A in comparison to control and hCG-treated cells. While control cells have a relatively faint cytoplasmic staining for StAR, hCG-treated cells are characterized by a diffuse and intense cytoplasmic staining pattern (Fig. 5a, c, e). By contrast, there was almost no signal for StAR in the activin-treated cells (Fig. 5a, c). The staining pattern for phospho-c-Jun was predominantly nuclear, which was further increased and decreased after treatment with hCG and activin-A, respectively (Fig. 5b, d, e).

### Culture of luteal GCs with activin-A in follicular fluid yields similar results

**Fig. 6:**
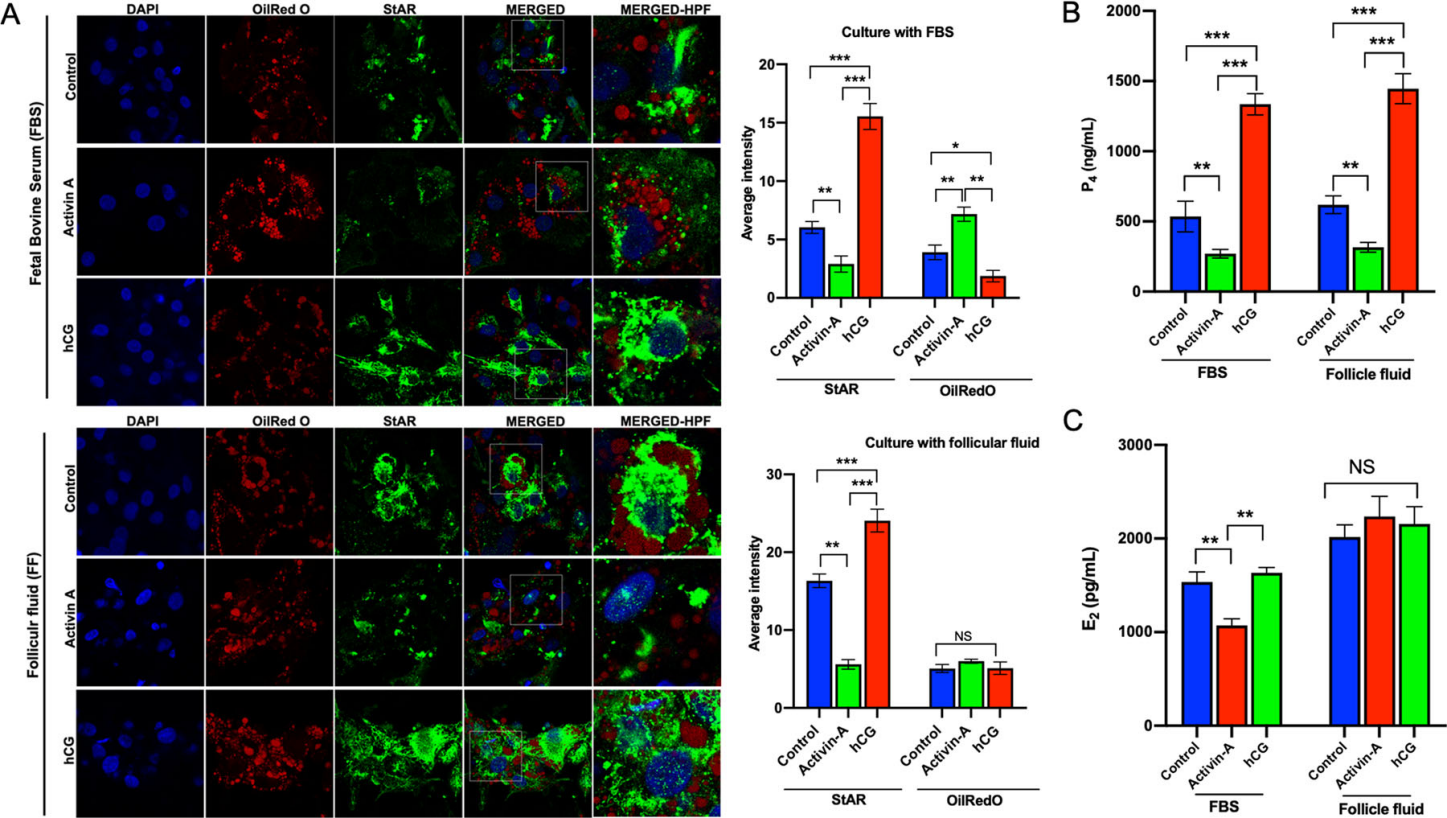
Comparison of the biological actions of activin-A on the expression of steroidogenic enzyme StAR on luteal granulosa cells cultured with fetal bovine serum (FBS) vs. follicular fluid (FF). Note the substantial decrease in the staining intensity of StAR and reduced P_4_ output after activin-A treatment similar to what it is observed in the cells cultured with FBS. hCG was associated significant increase in the StAR expression and P_4_ production, confirming its well-known luteotropic effects in both types of culture condition.

The cell culture experiments presented so far were carried out in the culture medium supplemented with FBS. We repeated the experiments with follicular fluid (FF) to see if activin-A will have the same suppressive effect on steroidogenesis when culture media is supplemented with androgen-rich FF instead of FBS. For this purpose, we first measured the concentrations of testosterone, E_2_, P_4_, hCG and lipids in the follicular fluids as shown in the Table 1. The mean concentrations of E_2_, P_4_, and testosterone were much higher in the FF samples compared to the FBS and culture media. Similar to the results of the experiment conducted with FBS, the expression of StAR was substantially decreased and increased in the cells after treatment with activin-A and hCG respectively, when they were cultured in culture medium with supplemented with FF (Fig. 6a). In line with that, P_4_ production of the cells decreased after activin-A treatment (Fig. 6b). Since the concentrations of E_2_ were extremely high in the FF samples (ranging from 141,935 to 305,000 pg/ml), it masked daily endogenous E_2_ production of the cells at baseline and after treatment with activin-A even when FF was used at 1–2% concentrations (Fig. 6c).

**Table 1:** The concentrations of sex steroids, lipids and hCG in the culture media, fetal bovine serum (FBS) and follicular fluid samples (FF).

	Culture media (DMEM-F12)	Fetal bovine serum (FBS)	Follicular fluid (FF)
Estradiol (pg/ml)	<5	<5	227,378 ± 86,133 (141,935–305,000)
Progesterone (ng/ml)	<0.1	<0.1	4,968 ± 1540 (3164–6452)
Testosterone (ng/ml)	<0.01	6.5 ± 0.7 (5.7–7.1)	498 ± 132 (289–879)
hCG (mIU/ml)	<0.1	<0.1	34 ± 26.7 (4.26–54.1)
Cholesterol (mg/dl) (Total)	4.5 ± 0.5 (4.0–5.1)	38.3 ± 4.5 (34–43)	14.6 ± 7.8 (5.9–21.1)
LDL (mg/dl)	4.4 ± 1.1 (3.6–5.7)	22.3 ± 3.2 (21–26)	4.16 ± 0.46 (3.9–4.7)

## Discussion

We showed in this study that activin-A downregulates the expression of steroidogenic enzymes StAR, P450 SCC, 3β-HSD, 17β-HSD, LH receptor, VEGF-A, and upregulates cyclin D1 and FSH receptor expression in human luteal granulosa cells. E_2_ and P_4_ production of the cells dramatically decreased after activin-A treatment. StAR is a rate-limiting enzyme in ovarian steroidogenesis that regulates cholesterol transfer from outer to inner mitochondrial membrane. P450 SCC catalyzes the conversion of cholesterol to pregnenolone and 3β-HSD converts pregnenolone to progesterone. 17β-HSD catalyzes the conversion of weak estrogen estrone (E_1_) to more potent estradiol (E_2_)^25^. Therefore, the suppression of these enzymes by activin-A resulted in decreased E_2_ and P_4_ production of the cells. Midcycle LH surge activates a intriguingly complex network of signaling cascades that will not only cause the rupture of the follicle and the release of the oocyte, but also triggers the release of many mediators of LH-induced signaling pathways. The latter is responsible for initiation of luteinization process and the formation of corpus luteum^26^. Luteinization process in fact, is characterized by upregulated expression of LH receptor, VEGF, increased vascularization and enhanced P_4_ output along with activation of inflammatory and proangiogenic responses^26^. Downregulation of FSH receptor and degradation of cyclin D1 (required for mitotic exit) are other hallmarks of luteinization event^23,26^. In accordance with this, we demonstrated in this study that activin-A downregulated the expression of steroidogenic enzymes, VEGF, LH receptor, and upregulated FSH receptor and cyclin D1 in human luteal GCs while hCG as a well-known luteotropic hormone exerted the opposite effect. Taken together, these actions of activin-A are consistent with reversal of luteinization phenotype or functional luteolysis in human luteal GCs.

Limited data is available regarding the physiological actions of activin-A and its receptor expressed on human luteal granulosa cells. While existing data is more consistent regarding the effects of activin-A on the expression of StAR and 3β-HSD and P_4_ production^18–22^, there is a controversy in literature regarding the effect of activin-A on aromatase expression and estrogen production in human luteal granulosa cells^21,22^. Our results are consistent with the findings of Dr. Jaffe’s team in 1991 that activin-A suppresses both P_4_ and E_2_ secretion in cultured human luteal granulosa cells^22^. In contrast to the findings of Chang et al.^21^, we did not observe any meaningful change in the aromatase expression in luteal granulosa cells when activin-A was administered at the same concentrations that they used. As a novel finding, we have shown in this study that activin-A downregulates JNK pathway along with steroidogenic enzymes in human luteal GCs. We recently showed that hCG-induced improvement in luteal function is associated with activation of JNK pathway. When JNK pathway was inhibited pharmacologically or knocked-down with siRNA luteal function was compromised and P_4_ production declined along with the expression of StAR and 3β-HSD in the cells^24^. It is worthwhile to note that the observed changes in the steroidogenic function of luteal GCs after JNK inhibition either pharmacologically or via siRNA are similar to those we observed after activin-A treatment. Taken together these data suggest that JNK signaling pathway play a pivotal role in the regulation of luteal function in human GCs by hCG and activin-A. In other words, luteotropic hCG improves luteal function by activating JNK pathway whereas activin-A reverses it by downregulating JNK pathway in human luteal GCs in vitro. StAR as a gatekeeper enzyme in steroidogenesis was upregulated and downregulated along with 3β-HSD, when JNK is activated by hCG and inhibited by activin-A respectively, in luteal GCs, indicating that StAR is closely associated with JNK pathway. In line with this, it was previously shown in a mouse Leydig cell tumor model that c-Jun binds to activator protein-1 (AP-1) motif in the promoter region of StAR and increases both StAR transcription and P_4_ synthesis^27^.

JNK as a member of mitogen-activated protein kinases (MAP kinases) is activated by a wide range of stimuli such as mitogenic agents, genomic damage, inflammation, and UV irradiation^28^. AP-1 transcription factors (Jun and Fos family) are the major downstream targets of JNK signaling pathway^28^. Limited data is available on the biological role of this pathway in human ovary. We previously documented the critical roles of JNK pathway in the regulation of FSH induced follicle growth and cell cycle progression of granulosa cells in mouse model^29,30^, in the pathogenesis of granulosa cell tumor in human^31^, and very recently in the hCG-induced improvement of luteal function in human luteal GCs^24^. Albeit not human a study on rat ovary and granulosa cells demonstrated that the expression patterns of Jun and Fos family members in response to FSH and LH were distinct. JunB, c-Jun, c-Fos, and Fra2 were rapidly but transiently induced by FSH in immature granulosa cells. JunD and Fra2 were induced by LH and maintained as granulosa cells terminally differentiated into luteal cells^32^.

Activin-A like the other members of TGF-β family act through the serine/threonine kinase receptors and Smad signaling^15^. However, previous studies documented that activin-A can also act via the mitogen-activated protein (MAP) kinase signaling pathway, which is generally induced in inflammatory states^33^. To the best of our knowledge, our study is the first one to identify a link between activin-A and JNK signaling in human granulosa cells. Activin-A and its receptors are widespread through female reproductive system and has diverse biological functions in the ovary, uterus, and during pregnancy^15^. Although biological functions of activins on granulosa cells were extensively studied, limited data is available regarding the biological roles of activin isoforms on human granulosa cells. Apparently, the action of this hormone varies depending upon whether or not the granulosa cells are luteinized or not. In nonluteinized human granulosa cells activin-A suppresses basal and FSH induced estradiol production^34^ and induces follicle growth by a direct mitogenic effect on granulosa cells by binding to its cognate receptors, and by enhancing FSH responsiveness of the follicle by upregulating the expression of FSH receptor and aromatase enzyme in human granulosa tumor cell line KGN^21,35–37^. Activin-rich intraovarian milieu characteristic of early follicular phase is later replaced by inhibin dominant environment during the processes of FSH induced further growth of a selected cohort of antral follicles and dominant follicle selection^36,38^. However, luteinized granulosa cells but not theca cells continue to express activin receptors in corpus luteum^39^. While serum activin-A level increases progressively during transition from mid to late luteal phase in human it is bounded and neutralized by follistatin, raising questions about its relative bioavailability for action on peripheral target cells^40^. Previous studies showed that activin-A is also found in the follicular fluid at the concentrations varying from <10 ng/ml to >200 ng/ml in patients undergoing IVF although no conclusive data exist regarding the biological role of this form of activin-A in luteal function or oocyte maturation^41–44^. Unfortunately we were not able to measure activin-A level in the follicular fluid samples that comes with mural luteal granulosa cells during oocyte retrieval procedure. However, activin-A was still able to downregulate the expression of StAR and reduce P_4_ output of the cells when they were cultured in androgen-rich follicular fluid. This observation is particularly important because granulosa cells lack 17-α hydroxylase and 17/20 lyase enzyme systems, which constitute the biological basis of the two-cell and two-gonadotrophins theory in human ovary^45–48^. Therefore, they rely on theca cell-derived androgens for conversion into estrogens by aromatase enzyme even after ovulation process luteinization event. Even though the FBS, that used in this study to supplement the culture medium, was a single batch and contained detectable amounts of testosterone, it was unknown until this experiment if activin-A would have the same suppressive effect on steroidogenesis when culture media is supplemented with androgen-rich FF instead of fetal bovine serum. Taken together activin-A consistently downregulates the expression of steroidogenic enzymes and reduces P_4_ production of granulosa cells after luteinization regardless of the origin of luteal granulosa cells (natural vs. stimulated IVF cycles), stimulation protocol and culture environment. This is the strength of our study.

Luteinization process and luteolysis are complex event that are poorly understood in human. While the former is characterized by a marked tissue remodeling, marked changes in intracellular signaling, gene regulations, extracellular matrix adhesion proteins, angiogenesis, and cell cycle regulators^3–5^ the latter can be defined as loss of function, decrease in P_4_ synthesis and output, tissue break-down, degradation of extracellular matrix proteins, infiltration of macrophages, and subsequent involution of the luteal structures and vessels and increased cell death^1,6^. There are certain clinical situations, where rapid regression of corpus luteum and reduction in sex steroid output and production of vasoactive and angiogenesis promoting factors like VEGF are of paramount importance such as ovarian hyperstimulation syndrome (OHSS). This syndrome is characterized by excess number of growing follicles >20 as a result of ovarian hyper-response to gonadotropin stimulation, enhanced ovarian neo-angiogenesis, supraphysiological serum levels of estrogen, increased vascular permeability and a fluid shift from intravascular space to the third space and edema due to elevated levels of vasoactive substances secreted by the ovaries such as VEGF, interleukins, tumor necrosis factor-alpha, and endothelin-1^49^. The current strategy to avoid this syndrome is to trigger ovulation with a GnRH agonist rather than hCG and freeze all embryos for transfer later because the latter has a longer half-life and promotes luteinization by upregulating the expression of the steroidogenic enzymes, VEGF, LH, and enhancing P_4_ output. hCG, which is either administrated exogenously for ovulation trigger; or produced endogenously by trophoblastic cells of the ensuing embryo if conception occurs deteriorates the clinical picture^50^. In this regard, activin-A as an hormone having opposing effects of hCG might at least help to reduce the severity of the syndrome if clinical studies confirm its beneficial effects in-vivo. In fact, the animal models of OHSS could be a good starting point before launching clinical studies.

Our study has a number of limitations. First, corpus luteum is a solid and dynamic organ composed of many different cell types and has as early, mid, and late phases. Mural luteal GC is only one of those cell types and represent early stage of the phase. Therefore, the biological actions of activin-A that we observed in these cells in vitro might not be true for the rest of the luteal phase. Second, luteal regression and luteolysis defined as the demise of corpus luteum is involves a complex interaction of immune cells with steroidogenic and stromal cells of the corpus luteum characterized by tissue break-down, degradation of extracellular matrix proteins, infiltration of macrophages, and subsequent involution of the luteal structures and vessels and increased cell death^1,6^. Thus, it is not a simple apoptotic death of the cells with subsequent reduction in steroidogenesis. Therefore, the observed in vitro actions of activin-A is not necessarily the same for real in vivo intraovarian environment.

## Conclusion

Activin-A appears to reverse luteinization phenotype in human luteal granulosa cells and JNK silencing through activin-A seems to be involved in the intricate mechanisms controlling the steroidogenic activity of corpus luteum in human. A better understanding of luteal functions at the molecular level might have a direct clinical impact. Identification of the molecular mechanisms in the formation of corpus luteum, luteinization, and luteolysis might facilitate the efforts to develop strategies against luteal phase pathologies commonly observed in assisted reproductive technologies.

## Materials and methods

This study was approved by the Institutional Review Board of Koc University (IRB# 2017.141.IRB2.069).

### Patients

Luteal granulosa cells were obtained from follicular aspirates during oocyte retrieval procedure in a total of 50 patients undergoing assisted reproduction treatment. Demographic and IVF characteristics of the patients are provided in Table 2. In stimulated IVF cycles, 225–300 IU recombinant FSH (Gonal F, Merck-Serono, Darmstadt, Germany) was initiated at early follicular phase to achieve multifollicular growth. In 32 patients, a GnRH antagonist (cetrorelix acetate, Cetrotide, Merck-Serono, Darmstadt, Germany) was started to prevent premature LH surge and ovulation when the dominant follicle reached 10–12 mm in size. In 12 patients, pituitary downregulation was induced using a GnRH agonist (leuprolide acetate, Lucrin, Abbott, IL, USA) from 7 days prior to the anticipated day of menstrual bleeding to the day of final oocyte maturation. In six natural cycle IVF patients, no ovarian stimulation was given and spontaneous follicle growth was achieved with endogenously produced FSH. In all cases, ovulation was triggered with 250 μg recombinant HCG (Ovitrelle; Merck-Serono, Darmstadt, Germany) when a leading follicle of ≥19 mm and two or more trailing follicles of ≥17 mm was recorded. Oocyte pick-up was performed 36 h after ovulation trigger.

**Table 2:** Demographic and IVF characteristics of the cycles (expressed as the mean ± SD).

	GnRH antagonist protocol (*n* = 32)	GnRH agonist protocol (*n* = 12)	Natural cycle IVF (*n* = 6)
Age (years)	32.4 ± 2.2	31.5 ± 2.1	36.3 ± 1.7
Duration of infertility (years)	2.7 ± 1.5	3.7 ± 1.7	4.5 ± 2.1
Mean gonadotropin dose (IU)	327 ± 31.0	337.5 ± 43.3	–
Duration of stimulation (days)	12.1 ± 1.3	11.7 ± 0.5	–
Peak estradiol E_2_ (pg/dl)	2790 ± 773.5	2822 ± 257.8.5	298 ± 31
Total number of oocytes retrieved	12.6 ± 2.5	12.7 ± 2.6	1.0
Metaphase-II oocytes	9.1 ± 2.1	10.5 ± 1.7	1.0

### Isolation and culture of human luteal GCs

Luteal GCs were isolated during oocyte retrieval procedure from follicular aspirates and cultured according to the procedures we previously described^16,24^. The cells were pooled for the experiment for most of the time only when the patients were comparable for age, infertility etiology, and stimulation protocol. If not, they were used individually rather than pooling should the number of cells recovered deemed sufficient for the experiments. Pooling not only provided us sufficient number of cells required for the experiments, but also allowed homogenous distribution of the cells among the experiments. However, the experiments were repeated in the cells coming from a single patient when the number of cells were sufficient.

The number of cells harvested varied among the protocols. Since there is only one naturally grown ovulatory follicle in the natural cycles, the number of cells obtained from these cycles was low (100,000–200,000 cells) compared to the stimulated cycles in which millions of cells could be harvested from each patient. In brief, recovered cells were cultured in 6-well format culture plates at a density of 500,000 cells per well using DMEM-F12 culture medium supplemented with 10% fetal bovine serum. For natural cycle, approximately 25,000 cells were plated for each well. For culture with follicular fluid (FF), the follicular aspirates obtained during oocyte retrieval procedure were spun down at 1000×*g* for 5 min. The supernatant was filtered through 0.22 µm filter and added to the culture medium at the indicated concentration. The cells were cultured at 37 °C and 5% CO_2_. One day after plating, the cells were treated with the hormones or chemicals at the indicated concentrations for 24 h. In brief, activin-A was used at 1–5–25–100 ng/ml, FSH 50 mIU/ml, and hCG 5 IU/ml concentrations.

### Chemicals and reagents

All cell culture materials were obtained from Gibco Inc. Recombinant Human Activin-A (GFH6) was purchased from Cell Guidance Systems. RepSox (#72392), a cell permeable, selective inhibitor of the TGF-β type 1 receptor (TGFβRI) ALK5 was obtained from Stemcell Technologies. Recombinant forms of FSH (Gonal-F) and hCG (Ovitrelle) was purchased from Merck Global (Darmstadt, Germany). SAPK/JNK Kinase Assay Kit (#8794, nonradioactive), Hoechst 33342 (#4082), anti c-Jun (#9165), anti-phospho-c-Jun^Ser73^ (#3270S), Anti-Phospho-SAPK/JNK^Thr183/Tyr185^ (#9251), Smad2 (#3122) and Phospho-SMAD2^Ser465/Ser467^ (#18338) antibodies were obtained from Cell Signaling. Oil Red O was purchased from Sigma Inc. (USA). All western blotting buffers and reagents were purchased from Bio-Rad. Anti-Vinculin Antibody (V9264) was purchased from Sigma-Aldrich. Mouse antihuman monoclonal antibodies were purchased from Santa Cruz Biotechnology for the detection of human 3β-HSD Type II (sc-100466), 17β-HSD type-I (sc-376719), StAR (sc-166821), and P450 SCC (CYP11A1, sc-292456). Aromatase (CYP19A1, ab34193) monoclonal mouse antibody was from Abcam Inc. YO-PRO®-1 Iodide (491/509) was obtained from Life Technologies. Texas Red™-X Phalloidin was obtained from Thermo Fisher Scientific.

### SAPK/JNK kinase assay

Endogenous JNK activity was measured by the nonradioactive assay kit instructions that described previously^31^. Briefly, the cells were harvested under nondenaturing conditions, lysed on ice and centrifuged. Then, 200 μl of cell/tissue lysates was incubated with 20 μl phospho-JNK rabbit monoclonal antibody linked to agarose beads to precipitate JNK enzyme. After addition of necessary buffers, c-Jun substrate and adenosine triphosphate, reaction mixture was incubated for 30 min at 30 °C, optimal reaction condition that allows c-Jun substrate to be phosphorylated by precipitated JNK. The reaction was stopped by adding 4× SDS sample buffer and the samples were loaded onto 10% polyacrylamide gel. Immunoblotting protocol was performed using monoclonal antibodies to measure JNK-induced phosphorylation of c-Jun substrate at Ser73.

### Viability assay

Live/dead cell assay was performed with YO-PRO (1 mM), a green-fluorescent carbocyanine nucleic acid stain absorbed by only apoptotic cells, whereas live cells are impermeant to it. Hoechst 33342 (1 mg/ml) was used for counterstaining. Live/dead cell imaging of the cells were undertaken under appropriate channels using an IF microscope (Olympus IX71, Japan).

### Gene expression analysis

RNA isolation was performed with Quick-RNA MicroPrep Kit (Zymo Research, Irvine, CA, USA) according to the manufacturer’s instructions. RNA was quantified with spectrophotometric read at 260 nm by Nanodrop 2000 (Thermo Fisher Scientific, MA, USA) and 500 ng cDNA was prepared by using M-MLV Reverse Transcriptase (Invitrogen). Quantitative real-time expressions of mRNAs of genes of interest were detected and compared by using Light Cycler 480 SYBR Green I Master (Roche, Germany). All primers that were used in this study are listed in the Table 3 below. The mean Cts and their SDs for each target gene were calculated from three technical replicates within the same quantitative RT-PCR (qRT-PCR) assay. We have used ΔΔCt method for relative quantitation of target genes^45,51^. The cDNA of the ovary used for the positive control of activin receptor expression in qRT-PCR experiment (Supplementary Fig. 1B) was already in our library and had been obtained from a 23 year patient undergoing laparoscopic excision of benign ovarian cyst within which some cortical piece was embedded.

**Table 3:** Primers used for qRT-PCR.

Gene	3′-Sequence-5′
GAPDH	F: ATGGAAATCCCATCACCATCTT
	R: CGCCCCACTTGATTTTGG
StAR	F: AAACTTACGTGGCTACTCAGCATC
	R: GACCTGGTTGATGATGCTCTTG
3β-HSD	F: GCCTTCAGACCAGAATTGAGAGA
	R: TCCTTCAAGTACAGTCAGCTTGGT
17β-HSD	F: TGGGGTCCACTTGAGCCTGAT
	R: TGCTGTGGGCGAGGTATTGG
CYP19A1 (Aromatase)	F: GGTCACCACGTTTCTCTGCT
	R: GCAAGCTCTCCTCATCAAACCA
CYP11A1 (P450 SCC)	F: CAGGAGGGGTGGACACGAC
	R: AGGTTGCGTGCCATCTCATAC
LH-R	F: TTGAACTGAGGTTTGTCCTCACCA
	R: GGCCTCAGGGTTGATGTAGAGC
FSH-R	F: TTTCAAGAACAAGGATCCATTCC
	R: CCTGGCCCTCAGCTTCTTAA
VEGF-A	F: TCGGGCCTCCGAAACCATGA
	R: TTCTGCCCTCCTCCTTCTGC
CYCLIN-D1	F: GCAATGACCCCGCACGATTT
	R: GTTGTTGGGGCTCCTCAGGT

### Hormone assays

E_2_, P_4_, testosterone, and hCG levels in culture media were determined by using electrochemiluminescence immunoassay “ECLIA” via the Roche Cobas-6000 analyzer (Roche, Mannheim, Germany). Lower detection limits of E_2_, P_4_, testosterone and hCG were 5.00 pg/ml (18.4 pmol/ml), 0.05 ng/ml (0.159 nmol/ml), 0.025 ng/ml (0.087 nmol/l) and 0.1 mIU/ml, respectively. The intra-assay CVs % of E_2_, P_4_, testosterone and hCG were, 2.4%, 2.3%, 2.9%, and 1.8%, respectively. The inter-assay CVs% of E_2_, P_4_, testosterone and hCG were 3.8%, 3.2%, 3.1%, and 4.6% respectively. Total cholesterol and LDL-C levels were measured by enzymatic, colorimetric methods, through the Roche Cobas-6000 analyzer (Roche, Mannheim, Germany). The intra-assay CVs% of total cholesterol and LDL-C and triglyceride were 1.1% and 0.7%, respectively. The interassay CVs% of total cholesterol and LDL-C were 1.6% and 2.3%, respectively.

### Western blotting

Proteins were extracted from samples using radioimmunoprecipitation assay buffer (Sigma-Aldrich). Protein concentration was quantified by standard BCA Assay method. Equal amounts of proteins for every sample (20 µg) were separated on SDS-PAGE and transferred onto Immuno-Blot® PVDF membrane using BioRad semi-dry blotting system. After blocking of nonspecific proteins, the membranes were incubated with primary antibodies overnight at 4 °C at dilutions instructed by the manufacturers. Following washing steps, membranes were incubated with HRP-conjugated secondary antibodies for 1 h at room temperature (RT). Anti-Vinculin was used the loading control. Following final washes and incubation with enhanced chemiluminescence (ECL), the signals on blots were visualized by the ChemiDoc MP Imaging System (Bio-Rad Laboratories, Inc).

### Immunofluorescence staining

Oil Red O (Sigma, USA) as a fat-soluble dye is used for staining of natural triglycerides and lipid droplets of the cells. Oil Red O working solution (0.5%) was prepared by boiling of 0.5 g Oil Red O in 100 ml 100% isopropanol. Cells were washed with Dulbecco’s phosphate-buffered saline (DPBS) and fixed with 4% paraformaldehyde (PFA) for 20 min at RT. Following washing with DPBS, cells rinsed at 60% isopropanol and stained with Oil Red O (Sigma, USA) for 20 min at RT. Subsequently, cells were rinsed by 60% isopropanol and running tap wate,r respectively and prepared for immunofluorescence staining steps. Permeabilization was performed in 0.2% Triton X-100 containing DPBS for 20 min at RT. Blocking of nonspecific epitopes was performed by incubation in Super Block (ScyTek, USA) medium for 20 min at RT. Thereafter, the cells were incubated with primary antibodies for overnight at 4 °C. Cells were washed three times with DPBS-Tween (0.01%), then incubated with secondary antibodies for 1 h at 37 °C. Cells were washed three times, then covered with Fluoroshield mounting medium with DAPI (Abcam, UK) and images were taken using a confocal microscope (Leica, DMI8).

### Statistical analysis

The samples size required for statistical significance and proper interpretation of the results was calculated according to the experimental methodologies used in the study as we previously described^24^. mRNA levels of the target genes used in the qRT-PCR assay (steroidogenic enzymes, LH and FSH receptor) and hormone levels are continuous variables therefore, expressed as the mean ± SD. ANOVA/ Bonferroni or Kruskal–Wallis/Dunn post-hoc tests were applied to compare the groups if data is parametric or nonparametric, respectively. The percentages of viable and apoptotic cells were compared between the groups using Fisher’ exact test. Significance level was set at 5% (*P* < 0.05). SPSS statistical program (version 22) was used to analyze the data.

## Supplementary information

The legends of the supplementary figures

Supplementary figure-1:

Supplementary figure-2:

Supplementary figure-3:

Supplementary figure-4:

Supplementary figure-5:
