# Supplementary material for: Terminal differentiation of human granulosa cells as luteinization is reversed by activin-A through silencing of Jnk pathway
Source: Cell Death Discov. 2020 Sep 23;6:93. doi: 10.1038/s41420-020-00324-9 (PMC7511402; doi:10.1038/s41420-020-00324-9)
Supplement: Supplementary file 1 — The legends of the supplementary figures [file 41420_2020_324_MOESM1_ESM.docx]

**SUPPLEMENTARY INFORMATION**

**Supplementary figure-1: Validation experiments that confirm the steroidogenic activity, the presence of activin receptors and their responsiveness to exogenously administered activin-A in human luteal granulosa cells in-vitro.**

The luteal granulosa cells are capable of maintaining their steroidogenic activity even under serum free culture condition. When fetal bovine serum (FBS) was added at 1 and 10% concentrations the steroidogenic activity of the cells increased in parallel with increasing FBS concentration (1A). Note the presence of lipid droplets used for steroidogenic function in the cells stained positive for OilRedO in these cells in light transmission and conventional (upper panel) and in comparison to fibroblast cell laser confocal immunofluorescence images (lower panel) (1B). The expression of activin receptors type 1B (ACVR1B), type 2A (ACVR2A) and type 2B (ACVR2B) were confirmed in the luteal granulosa cells with quantitative RT-PCR method. Ovarian cortex was used as a positive control (1C). The expression of phospho-Smad2^Ser465/Ser467^ was increased in a dose dependent manner after activin-A treatment at the indicated concentrations in western blotting. This effect was abolished when the cells were treated with activin-A and its receptor inhibitor (1D).

**Supplementary figure-2: Long-term steroidogenic activity of the luteal granulosa cells incubated with and without activin-A in culture.**

While control cells maintain their steroidogenic function and continued to produce E_2_ and P_4_ in relatively steady levels those treated with activin-A had significantly reduced E_2_ and P_4_ output when cultured up to 96hrs.

**Supplementary figure-3: Cell viability assay before and after activin-A treatment of luteal granulosa cells (GCs).** Intravital green fluorescent carbocyanine uptake assay (YO-PRO1 staining) did not reveal any significant differences between the vitality of the luteal GCs before and after activin-A treatment (97% vs. 96% respectively, p>0.05). Hoechst is chromatin stain.

**Supplementary figure-4: The effect of activin-A on the luteinization characteristics of the cells obtained from patients undergoing pure natural cycle (without gonadotropin stimulation)(upper panel) and stimulated IVF cycle using GnRH (gonadotropin releasing hormone) and gonadotropin stimulation (long protocol)(lower panel).**

Activin-A treatment was associated with down-regulation in the expression of steroidogenic enzymes and E_2_ and P_4_ production in luteal granulosa cells of different origin on qRT-PCR.

**Supplementary figure-5: The expression of gonadotropin receptors (FSH-R and LH-R), VEGF and cyclin-D1 before and after treatment with activin-A in luteal granulosa cells.**

The expression of LH-R and VEGF were down-regulated, FSH-R and cyclin D1 up-regulated after activin-A treatment. By contrast, luteotropic hCG had the opposite effect on qRT-PCR.
