# Supplementary figures and images for: Terminal differentiation of human granulosa cells as luteinization is reversed by activin-A through silencing of Jnk pathway
Source: Cell Death Discov. 2020 Sep 23;6:93. doi: 10.1038/s41420-020-00324-9 (PMC7511402; doi:10.1038/s41420-020-00324-9)

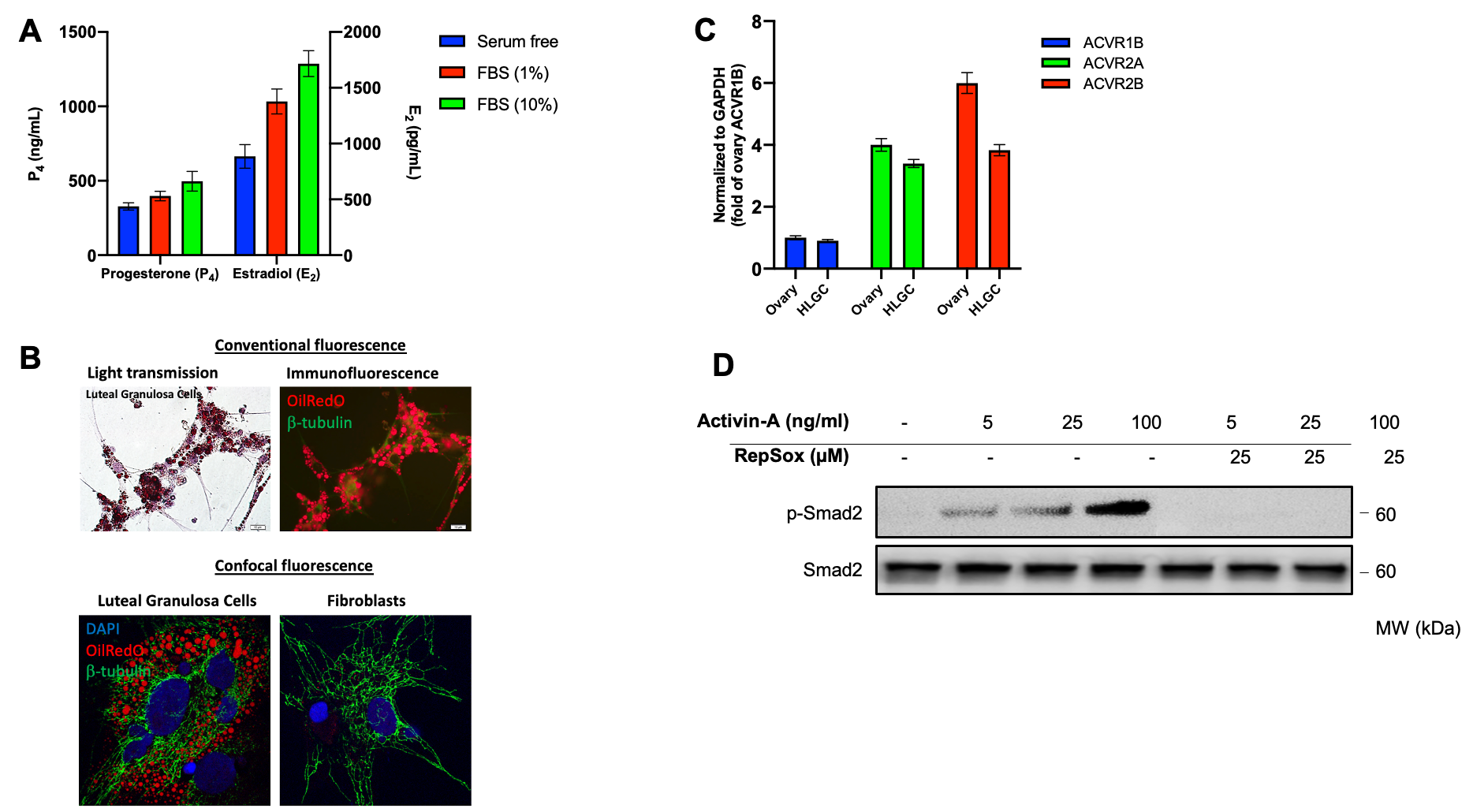

Supplement: Supplementary file 2 — Supplementary figure-1: [file 41420_2020_324_MOESM2_ESM.tif]

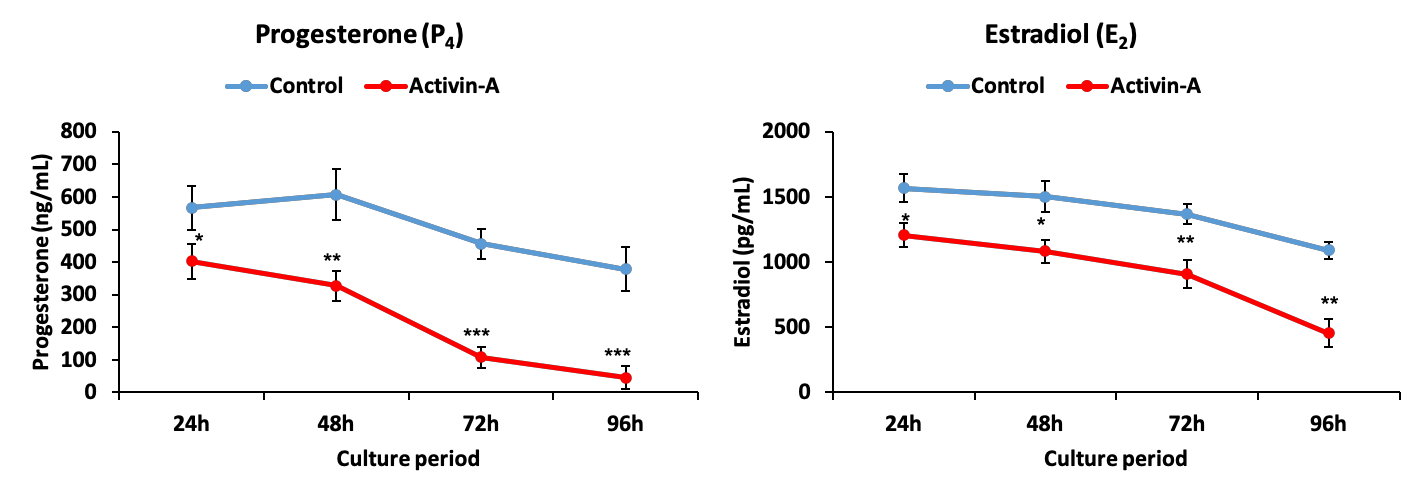

Supplement: Supplementary file 3 — Supplementary figure-2: [file 41420_2020_324_MOESM3_ESM.tif]

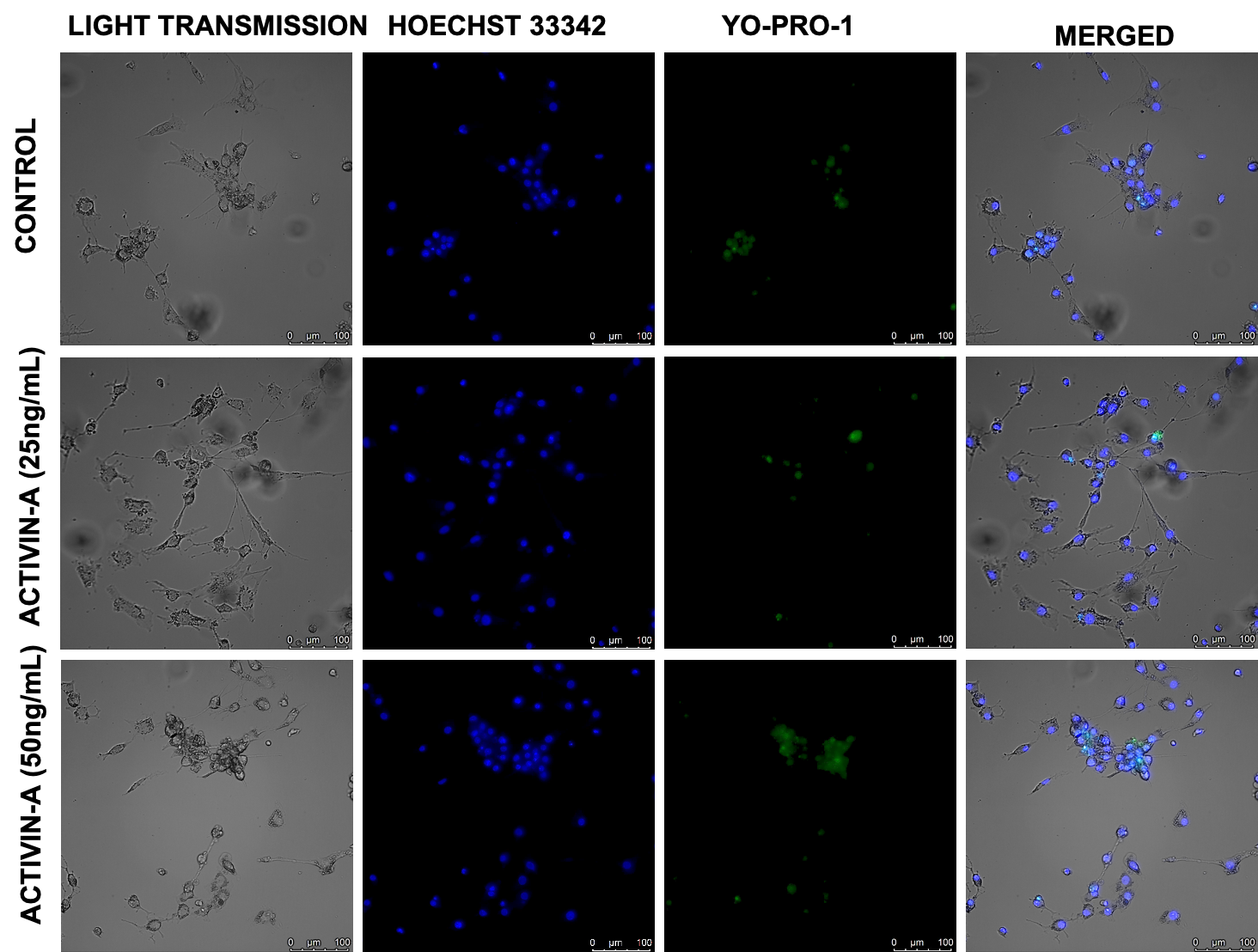

Supplement: Supplementary file 4 — Supplementary figure-3: [file 41420_2020_324_MOESM4_ESM.tif]

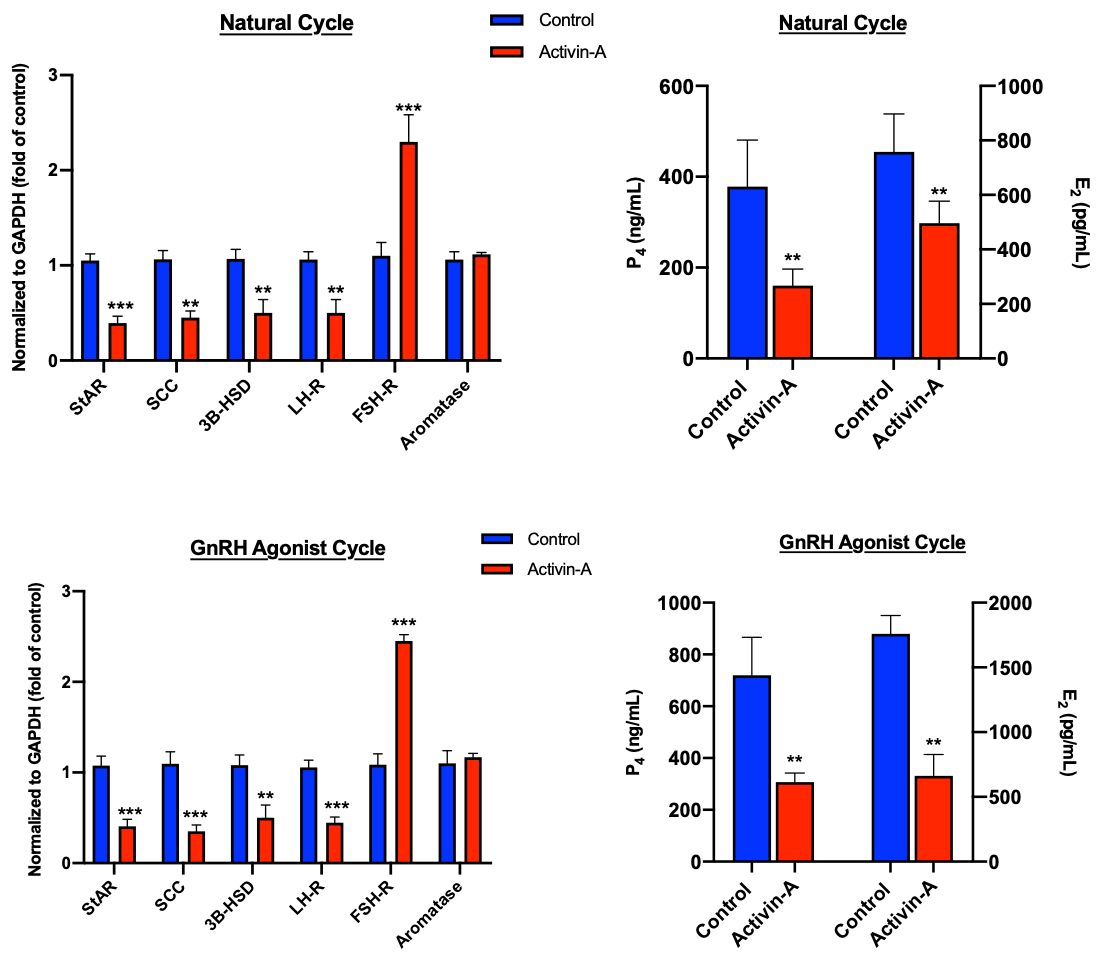

Supplement: Supplementary file 5 — Supplementary figure-4: [file 41420_2020_324_MOESM5_ESM.tif]

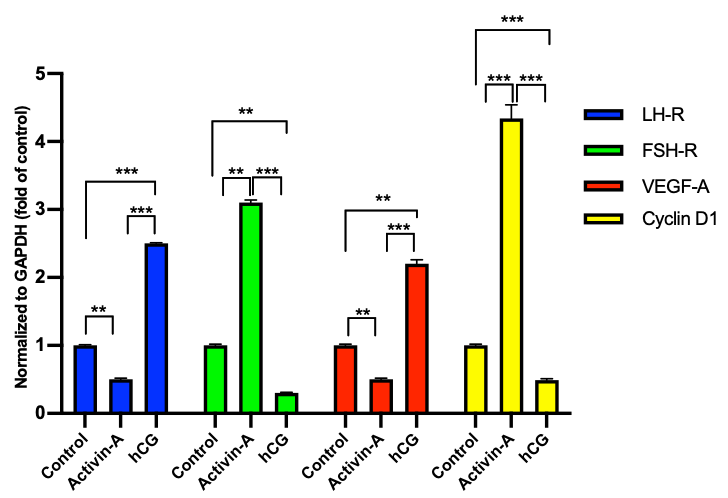

Supplement: Supplementary file 6 — Supplementary figure-5: [file 41420_2020_324_MOESM6_ESM.tif]
